# Development and Validation of a New Scoring System (Total Leishmania Score) for Dogs with *Leishmania infantum* Infection Including Clinical and Laboratory Parameters

**DOI:** 10.3390/pathogens15050517

**Published:** 2026-05-12

**Authors:** Julia C. Voelk, Melanie Kaempfle, Roswitha Dorsch, Vera Geisen, Ralf S. Mueller, Susanne K. Lauer, Yury Zablotski, Katrin Hartmann, Michèle Bergmann

**Affiliations:** LMU Small Animal Clinic, Centre for Clinical Veterinary Medicine, Ludwig Maximilian University of Munich (LMU Munich), 80539 Munich, Germanyhartmann@lmu.de (K.H.); michele.bergmann@lmu.de (M.B.)

**Keywords:** canine leishmaniosis, CanL, TLS, monitoring, disease severity, interobserver reliability, relapse, sensitivity to change

## Abstract

Canine leishmaniosis can cause a variety of signs. The detailed assessment of disease severity lacks a standardized, validated scoring system. This prospective study aimed to develop and validate an objective scoring system (“Total Leishmania Score”, TLS) combining clinical and laboratory parameters for monitoring dogs with *Leishmania (L.) infantum* infection. Fifty-one *L. infantum*-infected dogs were examined every 3 months over 1 year. Evaluations included physical examination, complete blood count, serum biochemistry, urinalysis including protein-to-creatinine ratio, and a *L. infantum* antibody Enzyme-Linked Immunosorbent Assay (ELISA). At each visit, 2 veterinarians applied the TLS, comprising 10 clinical and 8 laboratory parameters graded on a four-point severity scale (0–3) and weighted according to their estimated prognostic relevance values. Interobserver agreement was assessed using intraclass correlation coefficients (ICCs) and Bland–Altman analysis. Longitudinal changes were analyzed using robust linear mixed-effects models. In total, 488 scores were evaluated. Interobserver reliability was excellent (ICC: 0.998; CI_95%_: 0.997–0.998; *p* < 0.001) with no relevant systematic bias. Reliability remained excellent at all time points (ICC: 0.996–0.999). The TLS increased significantly before and during relapse (*p* < 0.001) and decreased significantly within 3 months after leishmanicidal treatment (*p* < 0.001). The TLS demonstrated excellent reliability and responsiveness, supporting its use for the longitudinal monitoring of dogs with leishmaniosis.

## 1. Introduction

Canine leishmaniosis is a vector-borne infectious disease caused by the protozoan parasite *Leishmania* (*L.*) *infantum*, transmitted by phlebotomine sandflies of the genus *Phlebotomus*. The disease is endemic throughout the Mediterranean area, including Southern Europe, the Middle East, and North Africa. Its geographic range has expanded considerably over recent decades, with increasing numbers of cases reported in Central and Northern Europe [1,2]. This northward spread is attributable to a combination of factors including climate change, increased travel and the importation of infected dogs, and the potential establishment of competent sandfly vectors in previously non-endemic regions [1]. As *L. infantum* is a zoonotic pathogen capable of infecting humans, particularly immunocompromised individuals, canine leishmaniosis represents not only a veterinary concern but also a significant health challenge [3].

Canine leishmaniosis is a chronic, multisystemic disease with a wide spectrum of clinical manifestations, ranging from subclinical infection to severe, potentially fatal illness [4]. The outcome of infection depends largely on the individual immune response of the host: while some dogs develop robust cell-mediated immunity and remain clinically healthy despite infection, others develop manifest disease [5]. Clinical signs typically reflect the involvement of multiple organ systems and can include dermatological lesions (scaling, alopecia, ulceration, and onychogryphosis), lymphadenopathy, weight loss, muscle atrophy, ocular lesions, epistaxis, and arthritis [3,6]. Laboratory abnormalities are equally diverse and commonly include anaemia, thrombocytopenia, leukopenia, hyperglobulinaemia, hypoalbuminaemia, elevated creatinine, and proteinuria [3]. The latter are of particular prognostic importance due to their association with immune-complex-mediated glomerulonephritis and progressive renal failure [7,8]. Thus, the disease shows considerable heterogeneity, which complicates the objective assessment of disease severity and monitoring of treatment response. The objective assessment of disease severity requires a detailed, logical, comparable, and reproducible classification of clinical and laboratory findings. Scoring systems provide a numerical quantification of disease severity, enabling the objective and detailed monitoring of changes over time; in contrast, classification systems assign patients to discrete disease stages based on predefined criteria. To standardize the assessment of disease severity in dogs with canine leishmaniosis, several scorings and classification systems for canine leishmaniosis have been proposed previously [4,6,9,10,11,12,13,14,15,16]. However, the existing scoring systems have notable limitations. While some systems distinguish only between the presence or absence of clinical signs without a further grading of severity, others lack important laboratory parameters essential for a comprehensive assessment of disease severity [12,13,14,15,16]. Moreover, the existing scoring systems have been criticized for assigning equal weight to each parameter, regardless of its prognostic relevance, and none of the scoring systems has been formally validated to date [17]. Furthermore, changes over time may not have been adequately assessed; this would be essential to reliably monitor disease progression and treatment response in individual patients.

Validation is a critical requirement for a scoring system to be reliably used in clinical practice and research as it ensures that the tool consistently measures the intended construct, yields reproducible results across different observers, and is sensitive enough to detect relevant changes over time [18]. Furthermore, validation strengthens the evidence base by enabling direct comparison between studies and supporting the reliability of meta-analyses.

Therefore, the aim of this prospective longitudinal study was to develop and validate a novel, comprehensive scoring system, the “Total Leishmania Score” (TLS), for dogs with *L. infantum* infection. The TLS was designed to integrate the most relevant clinical and laboratory parameters into a single quantitative score, with each parameter weighted according to its estimated prognostic relevance, thereby enabling the objective evaluation of disease severity and treatment outcome in canine leishmaniosis. The score was validated by assessing content validity, interobserver reliability, and longitudinal responsiveness.

## 2. Materials and Methods

### 2.1. Development of the Scoring System for Dogs with L. infantum Infection

The scoring system was developed by board-certified veterinarians in internal medicine (specifically infectiology, haematology, and urology), dermatology, and orthopaedics at the LMU Small Animal Clinic and was further refined after consultation with LeishVet members (see Section 2.3.2. Evaluation of Content Validity). It was based on the most common clinical and laboratory changes in dogs with leishmaniosis and on existing scores [3,14,15,17]. It included 10 clinical and 8 laboratory parameters: general condition, appetite, lymph node enlargement, dermatological lesions, ocular lesions, articular lesions, muscle atrophy, epistaxis, anaemia, thrombocytopenia, neutropenia, lymphopenia, renal azotaemia, hyperglobulinaemia, proteinuria, and the *L. infantum* antibody level. Parameters were graded from normal/not present (0) to mild (1), moderate (2), or severe (3) and weighted according to their estimated prognostic relevance (factor range: 1–40). Weighting factors were assigned based on current scientific knowledge and expert consensus among board-certified veterinarians in internal medicine (specifically infectiology, haematology, and urology), dermatology, and orthopaedics, with higher weighting factors allocated to parameters with a poorer prognostic relevance in canine leishmaniosis and lower weights to parameters with less prognostic impact [3,7,19,20]. Lymph node enlargement received the lowest weighting factors (factor 1 for mandibular, cervical, inguinal, and popliteal lymph nodes; factor 2 for any other lymph node), followed by muscle atrophy (factor 2), dermatological lesions and articular lesions (factor 2.5 each), and ocular lesions (factor 4 for conjunctivitis, blepharitis, and keratitis; factor 6 for uveitis). Epistaxis was assigned a weighting factor of 10, and haematological parameters (anaemia, thrombocytopenia, neutropenia, and lymphopenia) were each assigned a weighting factor of 15. General condition, appetite, hyperglobulinaemia, and the *L. infantum* antibody level were each assigned a weighting factor of 20. The highest weighting factors were assigned to renal azotaemia and proteinuria (factor 40 each). The adjusted score was calculated by multiplying the graded parameter score by the respective weighting factor. The TLS was defined as the sum of all adjusted scores (TLS range: 0–900) (Table 1). The TLS scoring sheet is available in Supplementary Materials (Table S1). Parameters were not included in the scores if deviations could be clearly attributed to other causes.

#### 2.1.1. Clinical Parameters

**Figure 1:**
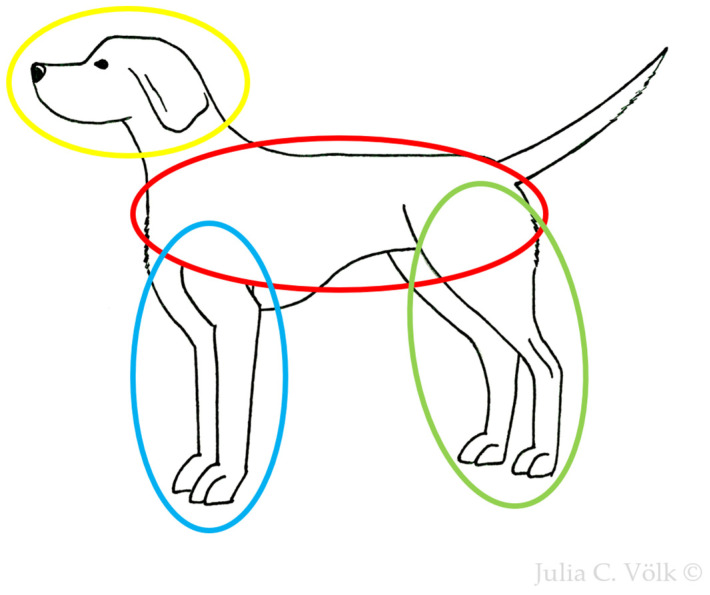
Simplified illustration of the body regions to be assessed individually for dermatological lesions (“papules/nodules/scaling/exfoliation/seborrheic dermatitis” and “ulcers”) in dogs with *Leishmania infantum* infection sectioned into head and pinnae (yellow-bordered), forelimb (blue-bordered), hindlimb (green-bordered), and torso (red-bordered) based on Plant et al. [21].

**Figure 2:**
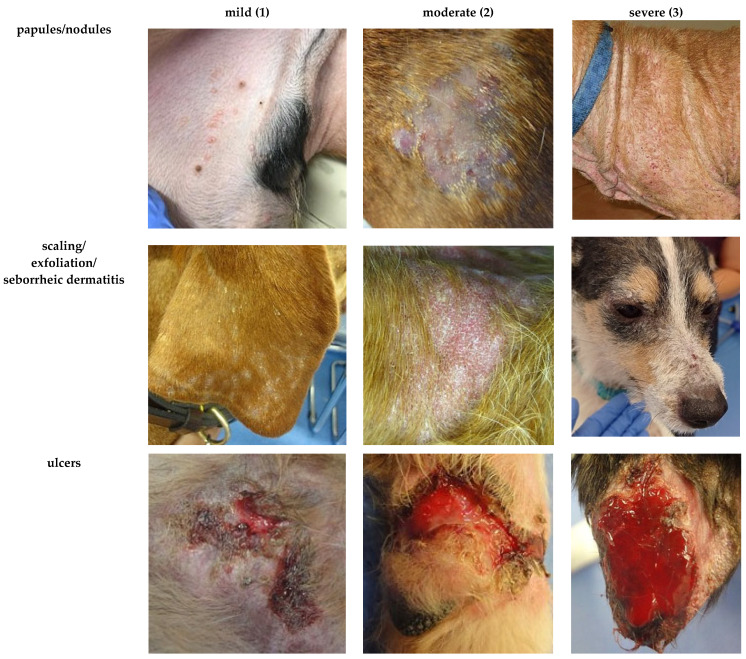
Examples for the gradation of the severity of the scored dermatological lesions as mild (1), moderate (2), or severe (3) in dogs with canine leishmaniosis.

**Figure 3:**
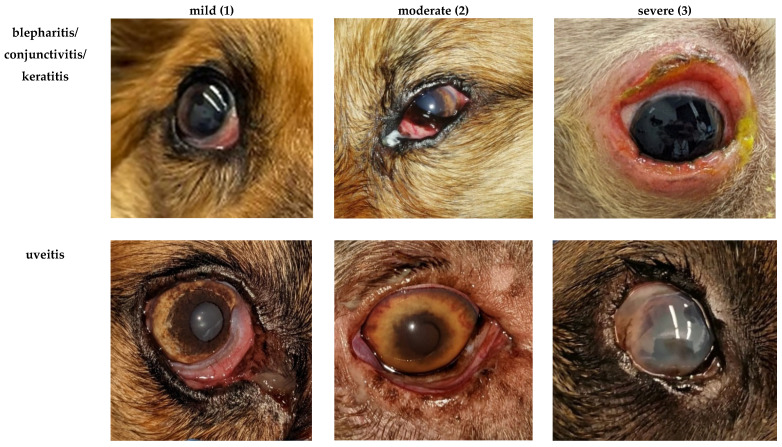
Examples for the gradation of the severity of the scored ocular lesions as mild (1), moderate (2), or severe (3) in dogs with canine leishmaniosis.

Our understanding of the dogs’ general condition was based on the owners’ reports and graded as unremarkable, mildly reduced, moderately reduced, or severely reduced. Scoring of the appetite was performed in accordance with an existing scoring system for dogs with leishmaniosis [15]. It considered each dog’s food intake during the last week according to the owner’s report and was graded as normal, mildly reduced (eating more than half of the usual portion), moderately reduced (eating less than half of the usual portion), or anorexia. Lymph node enlargement (of mandibular, cervical, inguinal, and popliteal lymph nodes) was graded based on the assessment of the scoring veterinarian (not present, mild, moderate, or severe enlargement). Enlargement of other lymph nodes (e.g., axillary or abdominal lymph nodes) could be assessed by grading the parameter “any other lymph nodes”. Evaluation of dermatological signs was based on the CADLI score regularly used for dogs affected by canine atopic dermatitis [21]. Different body regions were graded individually (Figure 1) for the most common dermatological lesions in canine leishmaniosis (“papules/nodules/scaling/exfoliation/seborrheic dermatitis” and “ulcers”). Figure 2 illustrates the grading of dermatological lesions based on representative cases with varying severity. Ocular lesions were considered individually for each eye (“blepharitis/conjunctivitis/keratitis” and “uveitis”) and graded as mild, moderate, or severe (Figure 3). The severity of joint lesions was assessed separately for each limb. The assessment of arthritis was based on the presence of joint swelling and/or pain on palpation or joint range of motion [23]. The muscle condition score (MCS) [22] was used to classify muscle atrophy. Grading of epistaxis was based on a previous publication as not present (no episodes of epistaxis during the last 3 months), sporadic (episodes <1/week, usually mild and self-limiting), frequent (episodes ≥1/week, moderate, occasionally requiring medical intervention), or persistent epistaxis (continuous, severe, requiring medical intervention) [12].

#### 2.1.2. Laboratory Parameters

Laboratory parameters were graded according to predefined thresholds for each parameter by internal medicine diplomates as shown in Table 1. Anaemia was graded as mild (haematocrit (HCT) < 37.3–30%), moderate (HCT < 30–20%), or severe (HCT < 20%) according to Meléndez-Lazo et al. (2018); thrombocytopenia as mild (platelets (PLT) < 148–110 × 10^9^/L), moderate (PLT < 110–60 × 10^9^/L), or severe (PLT < 60 × 10^9^/L); neutropenia as mild (neutrophils (NEU) < 2.95–1.5 × 10^9^/L), moderate (NEU < 1.5–1.0 × 10^9^/L), or severe (NEU < 1.0 × 10^9^/L); and lymphopenia as mild (lymphocytes (LYM) < 1.05–0.8 × 10^9^/L), moderate (LYM < 0.8–0.5 × 10^9^/L), or severe (LYM < 0.5 × 10^9^/L) [17]. Renal azotaemia was defined as elevated creatinine in conjunction with a urine specific gravity (USG) < 1.030 and was graded based on the International Renal Interest Society (IRIS) guidelines for staging of chronic kidney disease (CKD) for dogs as mild (creatinine > 125–250 µmol/L), moderate (creatinine > 250–440 µmol/L), or severe (creatinine > 440 µmol/L) azotaemia [24]. Hyperglobulinaemia was graded as mild (globuline (Glob) > 4.3–5 g/dL), moderate (Glob > 5–6 g/dL), or severe (Glob > 6 g/dL) according to Werner et al. (2004) [25]. Proteinuria was measured using the urine protein-to-creatinine ratio (UPC) and graded as mild (0.6–2), moderate (2.1–4), or severe (>4). In the presence of an active sediment (>5 leucocytes or >5 epithelial cells per high-power field, or presence of any bacteria and/or spermatozoon) or macroscopic haematuria, increased UPC results were not included in the score. For the detection of *L. infantum* antibodies, antibody levels were interpreted according to the laboratory’s specifications. Enzyme-linked immunosorbent assay (ELISA) levels between the cut-off and up to twice the cut-off were graded as mildly elevated and levels starting at twice the cut-off as moderately elevated while ELISA levels of approximately 3 times the cut-off were graded as severely elevated [26]. Accordingly, antibody levels were graded as mild (12.2–24.9 test unit (TU)), moderate (25–40 TU), or severe (>40 TU). This was consistent with the classification of IFAT titers by Sarquis et al. (2025): normal (<1/200), mildly elevated (1/200–1/400), moderately elevated (1/800–1/1600), and severely elevated (>1/1600) [27].

### 2.2. Study Population

The study obtained ethical approval from the ethical committee of the Centre for Clinical Veterinary Medicine of the LMU Munich (reference number 343-02-12-2022). The research adhered to German guidelines for prospective studies. Owners gave their written consent to participate prior to inclusion.

#### 2.2.1. Enrolment

Privately owned dogs that underwent regular rechecks as part of their ongoing canine *L. infantum* infection management were eligible for inclusion. Diagnosis had to be confirmed either by detection of antibodies via indirect immunofluorescence antibody tests (IFATs) or ELISA (each according to the respective laboratories’ cut-off values) within the last 3 months prior to inclusion, or by detection of *L. infantum* DNA by polymerase chain reaction (PCR). Owners were required to provide written consent to present their dogs every 3 months over a period of 1 year for routine monitoring.

At enrolment, a qualitative point-of-care (POC) test was performed to detect co-infections, especially antigen of *Dirofilaria (D.) immitis* and antibodies against *Ehrlichia (E.)* spp., *Anaplasma* spp., and *Borrelia burgdorferi* (SNAP^®^ 4Dx Plus, IDEXX Laboratories Inc., Westbrook, ME, USA). In addition, an abdominal ultrasound was performed to screen for comorbidities. ELISA testing for detection of *L. infantum* antibodies and *L.* spp. PCR from lymph node aspirates were also performed. Clinical evaluation comprised physical examination and scoring of clinical parameters by 2 independent veterinarians. Blood pressure analysis; complete blood count (CBC); a complete urinalysis, consisting of dip stick, urine specific gravity, sediment, and UPC; serum biochemistry; symmetrical dimethylarginine (SDMA); C-reactive protein (CRP); and antithrombin III (AT III) levels were examined.

Dogs with untreated co-infections with *E. canis*, *D. immitis*, and/or severe concomitant diseases were excluded. The TLS was completed by the 2 independent veterinarians after all laboratory results were obtained.

Overall, 51 dogs were included between July 2023 and July 2024. The study population included more females (*n* = 32; neutered: *n* = 25) than males (*n* = 19; neutered: *n* = 16) and more mixed-breed (*n* = 35) than purebred dogs (*n* = 16). The median age at the time of enrolment was 5.8 years (range: 1–12 years). With the exception of 1 dog (from Sri Lanka), all other dogs originated from endemic countries in Europe including Spain (*n* = 20), Greece (*n* = 13), Italy (*n* = 9), Croatia (*n* = 2), Portugal (*n* = 2), Romania (*n* = 1), Bosnia (*n* = 1), Bulgaria (*n* = 1), and Montenegro (*n* = 1) [28,29]. At the first study appointment, 37/51 (72.5%) dogs were graded as mildly affected by *Leishmania* infection (LeishVet stage I (*n* = 16) or stage II (substage IIa: *n* = 16, substage IIb: *n* = 5)) while 14/51 (27.5%) dogs were graded as severely affected (LeishVet stage III (*n* = 6) or stage IV (*n* = 8)) [4]. All dogs received allopurinol at the first study visit. One dog was switched from allopurinol to domperidone due to severe bilateral renal mineralization and partial obstruction. A total of 22 relapses were recorded, of which 14 were treated with miltefosine and 8 with meglumine antimoniate.

#### 2.2.2. Study Appointments

At all appointments, a detailed, structured history was obtained from the owner, specifically addressing general condition, appetite, epistaxis, and other anamnestical clinical abnormalities, to ensure consistent and systematic data collection.

At the follow-up appointments (months 3, 6, 9, and 12), physical examination and scoring of clinical parameters, measurement of blood pressure, CBC, complete urinalysis (dip stick, urine specific gravity, sediment, and UPC), serum biochemistry, SDMA, CRP, ELISA for detection of *L. infantum* antibodies, *L.* spp. PCR (lymph node aspirates), and ultrasound of the urinary tract were performed. At each study appointment, the TLS was completed by 2 independent veterinarians after all laboratory results were obtained.

#### 2.2.3. Laboratory Methods

Reference ranges were laboratory-specific, and each measurement followed standardized operating procedures. CBC was determined using an automated in-house analyzer (ProCyte Dx; IDEXX Laboratories Inc., Westbrook, ME, USA). Urine specific gravity was measured with an optical refractometer (RUR5-ATC; Hradec Králové, Czech Republic); urine dipstick testing was performed using IDEXX UA test strips analysed with the IDEXX UA Analyzer (IDEXX Laboratories Inc., Westbrook, ME, USA). Urine sediment was assessed using an automated in-house system (SediVue; IDEXX Laboratories Inc., Westbrook, ME, USA).

Fine-needle aspirates were obtained either from the popliteal lymph nodes or/and any other enlarged lymph nodes using a 22 gauge needle; the aspirate was expelled onto a glass slide and subsequently collected with a sterile cotton swab. Real-time PCR and further analyses (serum biochemistry, SDMA, CRP, AT III, UPC, ELISA) were performed at an external laboratory (IDEXX GmbH, Kornwestheim, Germany). Samples, including aliquots of serum, urine, citrate plasma, and the fine needle aspirate, were shipped on the day of collection in insulated packaging with a cooling pack and analysed the following day.

#### 2.2.4. Therapeutical Management

Treatment (supportive and/or with antileishmanial drugs) was performed heterogeneously based on presented alterations, disease progression, remission-free period and pre-treatment in accordance with standardized treatment regimes [4,30,31]. Allopurinol was administered at 10 mg/kg q12h PO in combination with a low-purine diet (allopurinol dose reduction was considered in case of urinary tract adverse events, e.g., severe mineralization of the urinary tract) [32,33]. In dogs that did not receive allopurinol, dietary nucleotides with active hexose correlated compound were administered according to package instructions. If indicated by an onset of new or worsening of existing signs, dogs received leishmanicidal treatment either with miltefosine 2 mg/kg orally once daily for 28 days or meglumine antimonate 100 mg/kg subcutaneously once daily for a minimum of 28 days. The decision for either drug was based on previously administered leishmanicidal treatment and the remission-free period [34]. In cases with suspected ocular involvement, dogs were referred to an ophthalmologic specialist to confirm diagnosis and adjust supportive therapy.

### 2.3. Prospective Validation of the TLS

#### 2.3.1. Pre-Testing

A preliminary pre-testing phase was conducted. The scoring system was applied once to 5 dogs with mild to severe *L. infantum* infection in order to assess whether the TLS reflected disease severity and was feasible for use in routine clinical practice.

#### 2.3.2. Evaluation of Content Validity

To assess content validity of the TLS, experts in canine leishmaniosis from the LeishVet Association were consulted regarding all aspects of the scoring system including the selected parameters, their weighting, and grading. Based on this assessment, minor adjustments were made where necessary.

#### 2.3.3. Evaluation of Reliability

To evaluate reliability, the TLS was applied to the 51 prospectively recruited dogs with *L. infantum* infection by 2 independent veterinarians within 2 h, each blinded to the other’s assessment. Veterinarian 1 was the primary researcher (corresponding author); veterinarian 2 was a veterinarian with comparable experience. The correlation between their scores was calculated to determine the agreement between the observers for each dog at every study appointment.

#### 2.3.4. Evaluation of Sensitivity to Change

Sensitivity to change was assessed based on changes in the dogs’ TLS values throughout the study course, specifically before and during relapse (onset of new or worsening of existing signs). This longitudinal analysis across predefined time points was designed to capture both disease progression and response to treatment, allowing assessment of whether changes in clinical and laboratory parameters were consistently reflected in the TLS.

### 2.4. Statistical Analysis

Each factor of the scoring system was analyzed separately to assess interobserver agreement using Fleiss’ kappa statistics. Kappa values were interpreted according to conventional thresholds: <0 = poor, 0–0.20 = slight, 0.21–0.40 = fair, 0.41–0.60 = moderate, 0.61–0.80 = substantial, or 0.81–1.00 = almost perfect agreement [35]. Confusion matrices were created to summarize how often both observers assigned identical or divergent scores.

Interobserver agreement between the 2 veterinarians was evaluated using the intraclass correlation coefficient (ICC) based on a 2-way model for absolute agreement and single measurements. ICC values were interpreted according to commonly defined thresholds (<0.50 poor, 0.50–0.75 moderate, 0.75–0.90 good, ≥0.90 excellent reliability) [36]. Agreement between observers was further evaluated using Bland–Altman analysis. Mean differences, corresponding 95% confidence intervals (CIs), and 95% limits of agreement (LoA) were calculated. A one-sample t-test was used to assess whether the mean bias differed significantly from 0. Bland–Altman analyses were performed for all observations combined and separately for each assessment (month 0, 3, 6, 9, and 12).

Analyses of the TLS before, during, and after relapse and treatment were performed using linear mixed-effects models. All mixed-effects models included individual animal as a random intercept to account for repeated measurements within animals. For the analysis of total score, veterinarian, month, and the veterinarian-by-month interaction were included as fixed effects. For the analysis of score, month, ownership, and the month-by-ownership interaction were included as fixed effects. Total score was also analyzed in a separate mixed-effects model with time point included as a fixed effect. For each model, conditional and marginal R^2^, the ICC, and the root mean square error (RMSE) were obtained. Estimated marginal means (EMMs) with 95% confidence intervals were calculated for each time point (0, 3, 6, 9, and 12 months) and for each observer. For pairwise contrasts, estimates, 95% confidence intervals, and *p*-values were reported. Model comparison was based on the Akaike Information Criterion (AIC), corrected AIC (AICc), and the Bayesian Information Criterion (BIC). All analyses were performed using R (Version 4.5.0) and the threshold for statistical significance was set at 5%.

## 3. Results

### 3.1. Dogs

Forty-five of the 51 dogs (88%) completed the entire study period. Four dogs died during the study course. Dogs that did not complete the study did not differ clinically from those completing the entire study period at the time of enrolment. One dog was euthanized due to progressive glomerulopathy with suspected glomerulonephritis caused by leishmaniosis with terminal renal failure. Two dogs died due to other reasons (road traffic accident and postoperative complications following surgery for brachycephalic obstructive airway syndrome). In 1 dog, the cause of death was unclear. The condition deteriorated suddenly and the dog died on the way to the veterinary clinic. Two dogs missed one of the 5 scheduled study appointments due to health-related circumstances (hospitalization of the dog at another clinic in one case and hospitalization of the owner in the other). In total, 244 study appointments were conducted, and each TLS was calculated by 2 veterinarians, resulting in 488 TLS values. Both veterinarians were from the same institution.

Of the 51 enrolled dogs, 18 had had at least one relapse during the study period, of which 4 dogs had 2 relapses, resulting in a total of 22 relapses. In 7 dogs, the TLS value noted 3 months before relapse was available for comparison, whereas in 11 dogs, the TLS value noted 3 months after relapse was available.

### 3.2. Scoring Results and Evaluation of Reliability

Detailed rating results are summarized in Table 2. The TLS values assessed by veterinarian 1 ranged from 0 to 335.0 (median TLS: 60.8), those by veterinarian 2 ranged from 0 to 328.5 (median TLS: 60.0).

Interobserver agreement data for individual TLS parameters is presented in Table 3. The evaluation of general condition and appetite, as well as all laboratory parameters, showed almost perfect agreement (κ = 1.0).

Agreement for lymph node enlargement showed moderate agreement for popliteal lymph node assessment (κ = 0.54), fair-to-moderate agreement for mandibular and cervical lymph node assessment (κ = 0.38–0.45), and slight agreement for inguinal lymph node assessment (κ = 0.15–0.21, accuracy = 99.6–99.7%). The assessment of any other lymph nodes (including enlarged axillary and abdominal lymph nodes) showed almost perfect agreement (κ = 0.87).

Agreement for dermatological lesions showed almost perfect agreement for ulcers of the head, torso, and forelimb (κ ≤ 0.86) whereas ulcers of the hindlimbs only showed moderate agreement (κ = 0.44). Scaling/exfoliation/seborrheic dermatitis/papules/nodules showed moderate-to-substantial agreement across all examined body regions (κ = 0.48–0.77).

Agreement for ocular lesions showed almost perfect agreement for uveitis in the right and left eye (κ = 0.93 and 0.82, respectively) and moderate agreement for blepharitis/conjunctivitis/keratitis in the right and left eye (κ = 0.70 and 0.73, respectively).

Agreement for arthritis and muscle atrophy ranged from moderate to almost perfect (κ = 0.54–0.85 and κ = 0.51–0.90 respectively).

Since only 1 dog had epistaxis, agreement resulted in a non-informative kappa value (κ ≈ 0.00; accuracy = 99.6%).

**Figure 4:**
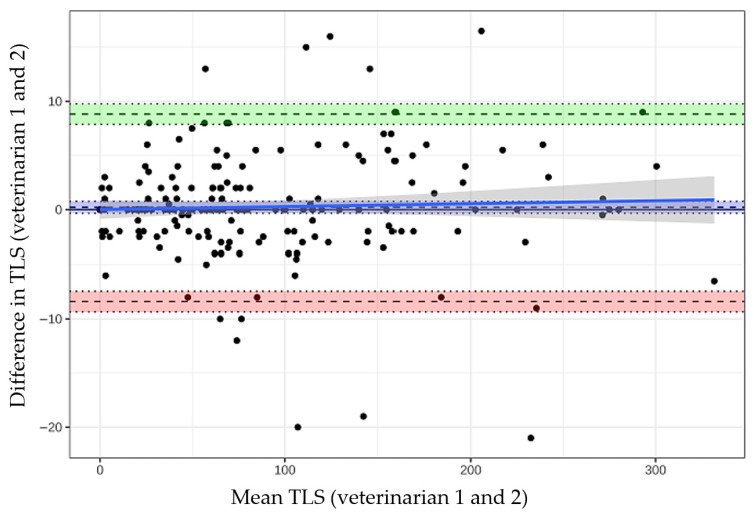
Interobserver agreement of the Total Leishmania Score (TLS) between 2 veterinarians assessed by Bland–Altman analysis. Each dot represents one dog. The black dashed lines represent the mean bias (0.22 points; 95% CI −0.33 to 0.77; *p* = 0.44) and the upper (+8.83 points) and lower (−8.39 points) 95% limits of agreement, respectively. The blue solid line with grey shaded area shows the linear regression of differences on means with its 95% confidence interval, indicating no clear evidence of proportional bias across the range of mean TLS values. The intraclass correlation coefficient was 0.998 (95% CI 0.997–0.998; *p* < 0.001).

Interobserver agreement of the total TLS values between the 2 veterinarians was excellent with an ICC of 0.998 (95% CI 0.997–0.998; *p* < 0.001), indicating nearly identical scoring between observers. Bland–Altman analysis showed no relevant systematic bias between veterinarians. The mean bias was 0.22 points (95% CI −0.33 to 0.77; *p* = 0.44), indicating no significant tendency for veterinarian 1 to score higher or lower than veterinarian 2. The 95% limits of agreement ranged from −8.39 to +8.83 points, meaning that approximately 95% of paired scores differed by less than ±9 points. The visual inspection of the Bland–Altman plot did not reveal proportional bias as score differences were equally distributed across the full range of the TLS (Figure 4).

Month-specific analyses confirmed consistently high interobserver agreement across all study appointments, with ICC values ranging from 0.996–0.999. Month-specific Bland–Altman analyses showed only small mean differences between observers (bias range −0.85 to + 0.98 points). Bias was not significantly different at months 0, 3, 6, and 9 (all *p* ≥ 0.14). At month 12, a small but statistically significant bias was observed, with veterinarian 1 scoring slightly higher on average (bias = 0.70 points; 95% CI 0.07–1.33; *p* = 0.030).

### 3.3. Evaluation of Sensitivity to Change

**Figure 5:**
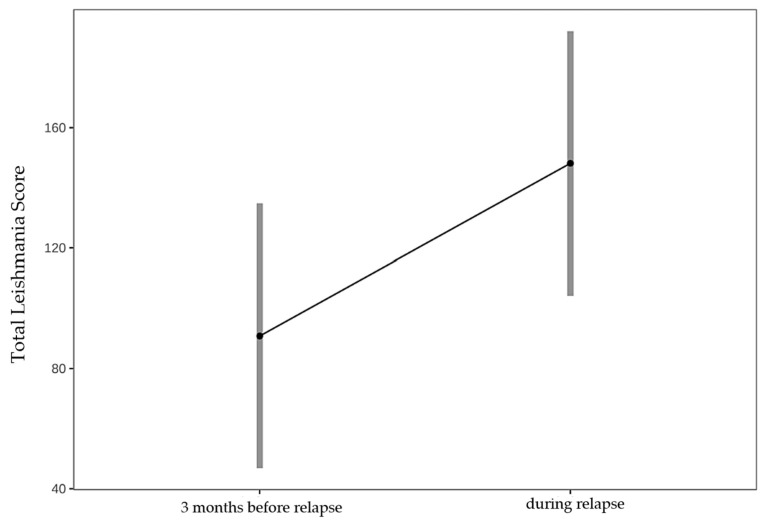
Comparison of the mean Total Leishmania Score (TLS) 3 months before and during relapse in 7 dogs with *Leishmania infantum* infection (14 paired examinations by 2 veterinarians). Mean TLS increased significantly from 90.8 (95% CI 46.8–135.0) before relapse to 148.1 (95% CI 104.1–192.0) during relapse (estimate −57.3, SE 14.3, z = −4.005, *p* < 0.001), as assessed by a linear mixed-effects model. Dots represent estimated marginal means; grey bars indicate 95% confidence intervals.

In 7 dogs with relapse, the mean TLS increased significantly from 90.8 (3 months before relapse) to 148.1 (during relapse) (*p* < 0.001) (Figure 5).

**Figure 6:**
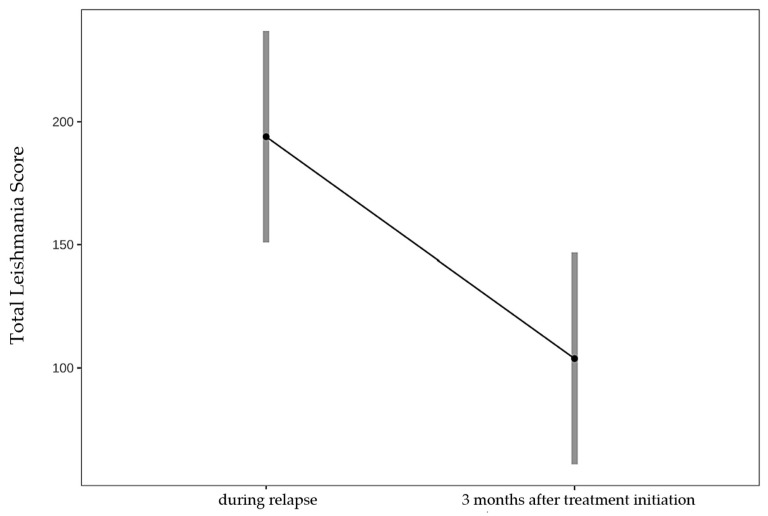
Comparison of the mean Total Leishmania Score (TLS) during relapse and 3 months later, after initiation of leishmanicidal treatment, in 11 dogs with *Leishmania infantum* infection (22 paired examinations by 2 veterinarians). Mean TLS decreased significantly from 194 (SE 22, 95% CI 151–237) during relapse to 104 (SE 22, 95% CI 61–147) 3 months after relapse (estimate 90.1, SE 15.5, z = 5.811, *p* <0.001), as assessed by a linear mixed-effects model. Dots represent estimated marginal means; grey bars indicate 95% confidence intervals.

In 11 dogs with relapse that were treated with leishmanicidal treatment, the mean TLS decreased significantly from 194 (during relapse) to 104 (3 months after relapse) (*p* < 0.001) (Figure 6).

## 4. Discussion

This study was, to the best of the authors’ knowledge, the first to validate a scoring system for dogs with *L. infantum* infection. The clinical validation of the TLS demonstrated excellent interobserver reliability, supporting its use for standardized assessment of disease severity. The high sensitivity to change of the TLS enables the detection of disease progression or remission in canine *Leishmania* spp. infections. The TLS therefore provides a standardized tool for use in clinical studies and supports comparability and meta-analysis.

Besides clinical parameters that had already been included in different previous scoring systems [12,13,14,15,16], the TLS additionally incorporated laboratory parameters inspired by the LeishVet staging [37] and the scoring of Paradies et al. (2010) [14]. Since clinical and laboratory parameters differ in their levels of estimated prognostic relevance [7,19,20,37,38], the TLS incorporated weighting factors for each parameter. This represents a distinguishing feature of the TLS as all previously published scoring systems for canine leishmaniosis applied equal weights to each parameter [12,13,14,15,16]. The benefit of weighting has been demonstrated in human medicine, where a weighted injury scoring system has shown better predictive performance than its unweighted counterpart [39]. The TLS further differs from other existing scores by providing a detailed assessment of each clinical parameter included; e.g., it assessed different dermatological lesions separately for each body region (Figure 1) and side, whereas others have assessed these as a single variable or by the percentage of body surface area affected [12,15]. This approach was chosen to more accurately assess the distribution and severity of lesions and to improve the ability to detect localized changes over time.

The TLS was based on established validated scoring instruments in terms of reliability and responsiveness such as the MCS [22] and the modified CADLI score [21], which have been successfully used in other studies [40,41,42,43]. In addition, reference images were used to standardize the grading of dermatological and ocular lesions. Visual reference tools have previously been shown to improve interobserver agreement [44]. These instruments likely contributed to the overall high interobserver reliability and sensitivity to change of the TLS. Only minor differences in interobserver agreement were noted for certain dermatological lesions (‘ulcers’ of the hindlimb or ‘scaling, exfoliation, seborrheic dermatitis, papules, nodules’ of the torso). Providing a broader set of reference images covering a wider range of clinical presentations per parameter and grading, e.g., ulcers at different anatomical locations such as the ear and the limbs, might further improve agreement in future applications. Moreover, the TLS included owner-assessed parameters (general condition and appetite), allowing insights into subtle changes that may not be adequately assessed by a veterinarian during a single clinical examination. The owner’s assessment represents a consistent data source independent of examiner variability [45].

Beyond confirming the overall structure and the weighting of the TLS, consultation with the expert panel resulted in the inclusion of epistaxis and an additional region (head and pinnae) for the assessment of muscle atrophy. Epistaxis, occurring in approximately 3.8–10% of infected dogs [6,17,46], can be induced by *Leishmania* spp. through different mechanisms, including direct mucosal invasion or secondary to thrombocytopenia, hyperglobulinaemia-induced thrombocytopathy, and/or vasculitis [47,48,49]. Muscle atrophy of the head, particularly affecting the temporalis and masticatory muscles, is reported in approximately 7.8–24.7% of infected dogs [17,46]. It can result from *Leishmania*-associated inflammatory myopathy, the direct invasion of myofibers by amastigotes, and/or IgG immune complex deposition [48,50].

Peripheral lymph node assessment was the least reliable parameter of the TLS (fair-to-moderate agreement), a finding that reflects the inherent subjectivity of palpation-based evaluation, which is strongly influenced by examiner experience [51]. Without objective measurements, veterinarians must rely on their impressions of size and firmness, which makes it harder to grade changes, particularly in cases of mild enlargement. Substantial discrepancies between palpation-based and imaging-based lymph node measurements have been well documented [52], underscoring the need for more standardized assessment approaches. A potential improvement for future applications would be to routinely measure lymph nodes when enlargement is suspected using a standardized scale adjusted for body size as normal lymph node dimensions vary considerably between different breeds and individually [53]. Importantly, however, although the two veterinarians scored slightly different, the influence on the TLS was relatively minor because lymph nodes were assigned low weighting factors based on the LeishVet classification (stage I) [37]. Even though lymph node enlargement has been shown to be a clinical sign raising suspicion of canine leishmaniosis [29], it does not necessarily correlate with either disease severity or the parasite burden within the lymph node [54].

Interobserver agreement for laboratory parameters was almost perfect (κ = 1.00). Grading thresholds for laboratory parameters were based on the reference intervals of the laboratory used in this study and should be adapted to the respective laboratory in other clinical settings.

In contrast to laboratory parameters of previous scoring systems, the TLS incorporated neutropenia and lymphopenia, which were observed in a study in *Leishmania*-infected dogs living in the same (non-endemic) area as the dogs in the present study. Furthermore, in this corresponding study, lymphopenia was shown to be prognostically relevant [7]. On the other hand, some parameters of previous scoring systems were intentionally omitted from the TLS. Urine specific gravity, polyuria, and polydipsia were omitted to avoid redundancy as owners were encouraged to ensure adequate water intake for the prevention of urolithiasis [55,56]. Total protein (TP) was not included due to the strong influence of albumin and globuline concentrations on TP values [57]. In canine leishmaniosis, globulines are typically elevated due to polyclonal B-cell activation and a marked humoral response [58] whereas albumin is often decreased as a consequence of proteinuria, a negative acute-phase reaction, or secondary to hyperglobulinaemia [59,60], parameters already included in the TLS. Including both TP and hypoalbuminemia would therefore not provide additional discriminatory value. As abdominal ultrasonography is not consistently available in routine clinical practice, intra-abdominal lymph nodes were not routinely assessed but could be scored under the parameter ‘any other lymph nodes’. Although several parameters were intentionally omitted from the TLS to minimize redundancy, a residual risk of redundancy among the remaining parameters cannot be fully excluded.

At month 12, a small but statistically significant bias between veterinarians 1 and 2 was identified, with veterinarian 1 consistently assigning slightly higher scores. However, given the range and variability of the TLS, this difference was negligible. No proportional bias was detected, indicating that interobserver differences were consistent across the score range and independent of disease severity. Although nearly 500 individual scores were evaluated, certain parameters, including epistaxis, specific gradings of ocular lesions, and some dermatological lesions occurred infrequently in the present study population. For certain parameters, including epistaxis and inguinal lymph node enlargement, low kappa values were observed despite high overall agreement, which is attributable to the low number of dogs showing these signs, a well-described prevalence effect of the kappa statistic. Accordingly, these values should be interpreted with caution and do not necessarily reflect poor interobserver reliability [61]. Future studies should evaluate the robustness of the TLS across diverse clinical settings and patient populations to further validate its broad applicability.

This study had several limitations. The study population was relatively small (*n* = 51), which can limit external validity. This is particularly relevant for sensitivity to change as data were available for only 7 dogs before relapse and 11 dogs after treatment. In addition, some parameters were infrequently observed, and certain severity categories were not represented. The presence of epistaxis in only 1 dog represents the major limitation, which is why kappa analysis did not yield valid results. Furthermore, the present study did not include outcome-based validation, such as the correlation of TLS values with survival or renal disease progression. Including such analyses in future studies with longer follow-up periods would help to further assess the clinical usefulness of the score. Parasite load was not quantified in this study, and the relationship between TLS values and parasite burden therefore remains unclear. Also, the influence of treatment as a confounding factor has not been determined. Weighting factors were based on expert consensus, and their prognostic relevance has not been established for all parameters. Data-driven approaches might further refine the weighting system in future studies. A further limitation is that no formal sensitivity analysis was performed to assess how alternative weighting schemes would influence the TLS. Although the weights were defined a priori based on estimated prognostic relevance, future studies with larger cohorts should evaluate the robustness of the TLS by comparing the current weighting system with alternative or unweighted scoring approaches.

## 5. Conclusions

The TLS is a promising and reliable tool for the standardized assessment of disease severity in canine leishmaniosis. The score incorporates routinely collected clinical and laboratory parameters, does not require specialized equipment, and is suitable for routine clinical practice. The TLS enables the objective monitoring of disease progression and treatment response and provides a standardized and objective scoring system for consistent outcome assessment, supporting the comparability of treatment studies across different study populations and settings. High interobserver reliability and sensitivity to change support its use in clinical and research settings.

## Figures and Tables

**Table 1:** Total Leishmania Score—Scoring system for dogs infected with *Leishmania infantum* to assess severity of infection based on the most common clinical and laboratory signs.

Parameter		0	1	2	3	Score (0–3) ^1^	Weighting Factor ^2^	Adjusted Score ^3^
clinical parameters								
general condition		normal	mildly reduced	moderately reduced	severely reduced		20	
appetite		normal	mildly reduced	moderately reduced	anorexia		20	
lymph node enlargement	right mandibular ln.	not present	mild	moderate	severe		1	
	left mandibular ln.	not present	mild	moderate	severe		1	
	right cervical ln.	not present	mild	moderate	severe		1	
	left cervical ln.	not present	mild	moderate	severe		1	
	right inguinal ln.	not present	mild	moderate	severe		1	
	left inguinal ln.	not present	mild	moderate	severe		1	
	right popliteal ln.	not present	mild	moderate	severe		1	
	left popliteal ln.	not present	mild	moderate	severe		1	
	any other ln.	not present	mild	moderate	severe		2	
skin ulcers *	head & pinna	not present	mild	moderate	severe		2.5	
	forelimb	not present	mild	moderate	severe		2.5	
	hindlimb	not present	mild	moderate	severe		2.5	
	torso	not present	mild	moderate	severe		2.5	
skin scaling/exfoliation/ seborrheic dermatitis/ skin papules/nodules *	head & pinna	not present	mild	moderate	severe		2.5	
	forelimb	not present	mild	moderate	severe		2.5	
	hindlimb	not present	mild	moderate	severe		2.5	
	torso	not present	mild	moderate	severe		2.5	
conjunctivitis/ blepharitis/keratitis	right eye	not present	mild	moderate	severe		4	
	left eye	not present	mild	moderate	severe		4	
uveitis	right eye	not present	mild	moderate	severe		6	
	left eye	not present	mild	moderate	severe		6	
arthritis	forelimb right	not present	joint swelling	pain on palpation/ROM	joint swelling + pain		2.5	
	forelimb left	not present	joint swelling	pain on palpation/ROM	joint swelling + pain		2.5	
	hindlimb right	not present	joint swelling	pain on palpation/ROM	joint swelling + pain		2.5	
	hindlimb left	not present	joint swelling	pain on palpation/ROM	joint swelling + pain		2.5	
muscle atrophy **	head	not present	mild	moderate	severe		2	
	forelimb right	not present	mild	moderate	severe		2	
	forelimb left	not present	mild	moderate	severe		2	
	hindlimb right	not present	mild	moderate	severe		2	
	hindlimb left	not present	mild	moderate	severe		2	
epistaxis		not present	sporadic	frequent	persistent		10	
laboratory parameters								
complete blood count	anaemia (HCT)	not present (ref: 37.3–61.7%)	mild (<37.3–30%)	moderate (<30–20%)	severe (<20%)		15	
	thrombocytopenia (PLT)	not present (ref: 148–484 × 10^9^/L)	mild (<148–110 × 10^9^/L)	moderate (<110–60 × 10^9^/L)	severe (<60 × 10^9^/L)		15	
	neutropenia (NEU)	not present (ref: 2.95–11.64 × 10^9^/L)	mild (<2.95–1.5 × 10^9^/L)	moderate (<1.5–1.0 × 10^9^/L)	severe (<1.0 × 10^9^/L)		15	
	lymphopenia (LYM)	not present (ref: 1.05–5.1 × 10^9^/L)	mild (<1.05–0.8 × 10^9^/L)	moderate (<0.8–0.5 × 10^9^/L)	severe (<0.5 × 10^9^/L)		15	
serum biochemistry	renal azotaemia (creatinine)	not present (ref: 44–125 µmol/L)	mild (>125–250 µmol/L)	moderate (>250–440 µmol/L)	severe (>440 µmol/L)		40	
	hyperglobulinaemia (Glob)	not present (ref: 2.4–4.3 g/dL)	mild (>4.3–5 g/dL)	moderate (>5–6 g/dL)	severe (>6 g/dL)		20	
others	proteinuria (UPC)	not present (0–0.5)	mild (0.6–2)	moderate (2.1–4)	severe (>4)		40	
	antibody level (ELISA)	normal (0–12 TU)	mildly elevated (12.1–24.9 TU)	moderately elevated (25–40 TU)	severely elevated (>40 TU)		20	
Total Leishmania Score = sum of all adjusted scores (range: 0—900)								

ln: lymph node; ROM: range of motion; L: liters; µmol: micromole; g: gram; dL: deciliter; HCT: haematocrit; PLT: platelets; NEU: neutrophils; LYM: lymphocytes; Glob: globuline; UPC: urine protein-to-creatinine ratio; ELISA: enzyme-linked immunosorbent assay; TU: test unit. ^1^ score; veterinarians were required to grade each parameter on a scale from 0 to 3 (0: normal/not present; 1: mild; 2: moderate; 3: severe). ^2^ weighting factors for existing signs according to their estimated prognostic relevance. ^3^ adjusted score = “score” of the graded parameters multiplied by the respective “weighting factor”. * grading based on the score for canine atopic dermatitis [21]. ** grading based on the muscle condition score [22].

**Table 2:** Frequency distribution of Total Leishmania Score (TLS) parameter ratings across score categories (0 = not present/normal, 1 = mild, 2 = moderate, 3 = severe) for all 488 assessments performed independently by 2 veterinarians throughout the study period.

Parameter			0	1	2	3
clinical parameters	general condition		474 (97.1)	14 (2.9)	0 (0)	0 (0)
	appetite		486 (99.6)	2 (0.4)	0 (0)	0 (0)
	lymph node enlargement	right mandibular ln.	392 (80.4)	70 (14.3)	24 (4.9)	2 (0.4)
		left mandibular ln.	398 (81.6)	68 (13.9)	22 (4.5)	0 (0)
		right cervical ln.	452 (92.7)	28 (5.7)	8 (1.6)	0 (0)
		left cervical ln.	452 (92.7)	26 (5.3)	10 (2.1)	0 (0)
		right inguinal ln.	484 (99.2)	4 (0.8)	0 (0)	0 (0)
		left inguinal ln.	484 (99.2)	4 (0.8)	0 (0)	0 (0)
		right popliteal ln.	316 (64.8)	132 (27.0)	38 (7.8)	2 (0.4)
		left popliteal ln.	316 (64.8)	140 (28.7)	28 (5.7)	4 (0.8)
		any other ln.	472 (96.8)	8 (1.6)	6 (1.2)	2 (0.4)
	skin ulcers *	head & pinna	450 (92.3)	22 (4.5)	8 (1.6)	8 (1.6)
		forelimb	476 (97.6)	2 (0.4)	6 (1.2)	4 (0.8)
		hindlimb	478 (98.0)	10 (2.0)	0 (0)	0 (0)
		torso	484 (99.2)	4 (0.8)	0 (0)	0 (0)
	skin scaling/exfoliation/ seborrheic dermatitis/ skin papules/nodules *	head & pinna	374 (76.7)	108 (22.1)	4 (0.8)	2 (0.4)
		forelimb	458 (93.9)	30 (6.1)	0 (0)	0 (0)
		hindlimb	456 (93.4)	32 (6.6)	0 (0)	0 (0)
		Torso	430 (88.1)	54 (11.1)	2 (0.4)	2 (0.4)
	conjunctivitis/ blepharitis/keratitis	right eye	445 (91.2)	37 (7.6)	6 (1.2)	0 (0)
		left eye	446 (91.4)	32 (6.6)	10 (2.0)	0 (0)
	uveitis	right eye	474 (97.1)	0 (0)	0 (0)	14 (2.9)
		left eye	472 (96.7)	0 (0)	2 (0.4)	14 (2.9)
	arthritis	forelimb right	464 (95.2)	10 (2.0)	10 (2.0)	4 (0.8)
		forelimb left	464 (95.1)	14 (2.9)	8 (1.6)	2 (0.4)
		hindlimb right	464 (95.1)	12 (2.5)	10 (2.0)	2 (0.4)
		hindlimb left	462 (94.8)	10 (2.0)	10 (2.0)	6 (1.2)
	muscle atrophy **	head	464 (95.1)	22 (4.5)	2 (0.4)	0 (0)
		forelimb right	466 (95.5)	18 (3.7)	4 (0.8)	0 (0)
		forelimb left	472 (96.7)	14 (2.9)	2 (0.4)	0 (0)
		hindlimb right	456 (93.5)	24 (4.9)	6 (1.2)	2 (0.4)
		hindlimb left	454 (93.1)	26 (5.3)	6 (1.2)	2 (0.4)
	epistaxis		486 (99.6)	2 (0.4)	0 (0)	0 (0)
laboratory parameters	complete blood count	anaemia (HCT)	392 (80.3)	78 (16.0)	18 (3.7)	0 (0)
		thrombocytopenia (PLT)	464 (95.1)	16 (3.3)	6 (1.2)	2 (0.4)
		neutropenia (NEU)	408 (83.6)	80 (16.4)	0 (0)	0 (0)
		lymphopenia (LYM)	440 (90.2)	26 (5.3)	20 (4.1)	2 (0.4)
	serum biochemistry	renal azotaemia (creatinine)	454 (93.1)	26 (5.3)	6 (1.2)	2 (0.4)
		hyperglobulinaemia (Glob)	422 (86.5)	34 (7.0)	18 (3.7)	14 (8.2)
	others	proteinuria (UPC)	366 (75.0)	64 (13.1)	18 (3.7)	40 (8.2)
		antibody level (ELISA)	96 (19.7)	114 (23.4)	92 (18.9)	186 (38.0)

ln: lymph node; HCT: haematocrit; PLT: platelets; NEU: neutrophils; LYM: lymphocytes; Glob: globuline; UPC: urine protein-to-creatinine ratio; ELISA: enzyme-linked immunosorbent assay; * grading based on the score for canine atopic dermatitis [21]; ** grading based on the muscle condition score [22].

**Table 3:** Interobserver agreement between 2 veterinarians for individual TLS parameters, including Cohen’s kappa (κ), overall agreement (accuracy), 95% confidence intervals, and McNemar’s test.

Parameter			κ	Accuracy	95% CI	McNemar’s *p* Value
clinical parameters	general condition		1.00	1.00	(0.99–1.00)	-
	appetite		1.00	1.00	(0.99–1.00)	-
	lymph node enlargement	right mandibular ln.	0.46	0.83	(0.78–0.88)	-
		left mandibular ln.	0.38	0.82	(0.76–0.86)	-
		right cervical ln.	0.46	0.91	(0.86–0.94)	0.06
		left cervical ln.	0.51	0.92	(0.87–0.95)	0.02
		right inguinal ln.	0.21	0.97	(0.94–0.99)	0.13
		left inguinal ln.	0.16	0.96	(0.93–0.98)	0.03
		right popliteal ln.	0.54	0.78	(0.72–0.83)	-
		left popliteal ln.	0.54	0.78	(0.72–0.83)	-
		any other ln.	0.90	0.99	(0.97–1.00)	-
	skin ulcers	head & pinna	0.86	0.98	(0.95–0.99)	-
		forelimb	1.00	1.00	(0.99–1.00)	-
		hindlimb	0.44	0.98	(0.95–0.99)	-
		torso	0.67	1.00	(0.98–1.00)	1.00
	skin scaling/exfoliation/ seborrheic dermatitis/ skin papules/nodules	head & pinna	0.77	0.92	(0.87–0.95)	-
		forelimb	0.53	0.95	(0.92–0.98)	-
		hindlimb	0.61	0.95	(0.92–0.98)	-
		torso	0.48	0.90	(0.86–0.94)	-
	conjunctivitis/ blepharitis/keratitis	right eye	0.70	0.95	(0.92–0.98)	0.54
		left eye	0.73	0.96	(0.92–0.98)	0.55
	uveitis	right eye	0.93	1.00	(0.98–1.00)	1.00
		left eye	0.82	0.99	(0.97–1.00)	-
	arthritis	forelimb right	0.68	0.98	(0.95–0.99)	-
		forelimb left	0.85	0.99	(0.97–1.00)	-
		hindlimb right	0.58	0.97	(0.94–0.99)	-
		hindlimb left	0.54	0.96	(0.93–0.98)	-
	muscle atrophy	head	0.90	0.99	(0.97–1.00)	-
		forelimb right	0.51	0.96	(0.93–0.98)	-
		forelimb left	0.64	0.98	(0.95–0.99)	-
		hindlimb right	0.68	0.96	(0.93–0.98)	-
		hindlimb left	0.64	0.95	(0.92–0.98)	-
	epistaxis		0.00	1.00	(0.98–1.00)	1.00
laboratory parameters	complete blood count	anaemia (HCT)	1.00	1.00	(0.99–1.00)	-
		thrombocytopenia (PLT)	1.00	1.00	(0.99–1.00)	-
		neutropenia (NEU)	1.00	1.00	(0.99–1.00)	-
		lymphopenia (LYM)	1.00	1.00	(0.99–1.00)	-
serum biochemistry	renal azotaemia (creatinine)	1.00	1.00	(0.99–1.00)	-
		hyperglobulinaemia (Glob)	1.00	1.00	(0.99–1.00)	-
	others	proteinuria (UPC)	1.00	1.00	(0.99–1.00)	-
		antibody level (ELISA)	1.00	1.00	(0.99–1.00)	-

κ: Cohen’s kappa coefficient; CI: confidence interval; accuracy: proportion of identical ratings between observers; McNemar’s *p*: *p*-value of McNemar’s test; ln: lymph node; HCT: haematocrit; PLT: platelets; NEU: neutrophils; LYM: lymphocytes; Glob: globuline; UPC: urine protein-to-creatinine ratio; ELISA: enzyme-linked immunosorbent assay; Dashes (-) indicate that the respective category was not present or that the metric could not be calculated.

## Data Availability

The raw data supporting the conclusions of this article will be made available by the authors on request.

## Supplementary Materials

The following supporting information can be downloaded at: https://www.mdpi.com/article/10.3390/pathogens15050517/s1, Table S1: Total Leishmania Score.
